# Drug-Induced Liver Failure Following Topical Minoxidil: A Case Report

**DOI:** 10.1155/crgm/1040092

**Published:** 2025-11-04

**Authors:** Katrin Premke, Matthias Kästner, Carlos Fritzsche, Andreas Erbersdobler, Micha Loebermann

**Affiliations:** ^1^Department of Tropical Medicine and Infectious Diseases, Rostock University Medical Center, Rostock, Germany; ^2^Department of Pathology, Rostock University Medical Center, Rostock, Germany

## Abstract

**Introduction:**

Drug-induced liver failure (DILI) is one of the causes of acute liver injury (13%–20%), encompassing both predictable and idiosyncratic reactions. While the latter are rare, it can lead to severe liver damage in vulnerable patients. Although topical minoxidil is a widely used treatment for androgenetic alopecia, cases of liver failure linked to its use are extremely uncommon and have not been widely described. This case report discusses the potential for severe liver injury following topical minoxidil application.

**Case Presentation:**

A 21-year-old female with no significant medical history presented with jaundice, elevated liver enzymes, and impaired liver function three and a half weeks after starting topical minoxidil for alopecia areata. Laboratory tests revealed significantly elevated transaminases, bilirubin, and disturbed coagulation. A liver biopsy revealed centrilobular necrosis, which indicated drug-induced liver damage. After discontinuation of minoxidil, the patient's condition improved rapidly, with a marked decrease in liver enzymes and disappearance of clinical symptoms.

**Conclusions:**

This is the first evidence of severe drug-induced liver failure associated with topical minoxidil. It underscores the potential for hepatotoxicity even with over-the-counter drugs that are thought to have minimal systemic absorption. The rapid improvement after discontinuation of the drug and exclusion of differential diagnoses suggests a causal relationship. In cases of severe liver failure of unclear origin, all substances should be considered in the case history.

## 1. Introduction

Drug-induced liver injury (DILI) is a significant cause of acute liver failure ranging from mild enzyme alterations to severe hepatotoxicity [1, 2]. The incidence of DILI is estimated at 20 cases per 100.000 individuals annually and accounts for 11% of acute liver failure cases in the United States [3]. Idiosyncratic DILI is particularly common, often linked to antimicrobials, herbal supplements, and anticancer drugs, and can present with various symptoms, including jaundice and coagulopathy [4]. Risk factors for DILI include high drug dosage, lipophilicity, and the extent of hepatic metabolism, though the role of host factors remains unclear [3]. The role of liver biopsy is crucial for understanding the underlying pathogenesis and guiding therapy [5]. Management primarily involves discontinuation of the offending drug and supportive care.

Originally developed as an antihypertensive, minoxidil is now widely used for hair loss treatment, particularly androgenetic alopecia [6], and has shown promise in treating alopecia areata [7]. Its mechanism involves vasodilation and potassium channel activation, enhancing blood flow to hair follicles [8]. Studies show cosmetically acceptable regrowth in about one-third of patients after one year [9]. Scalp irritation and contact dermatitis are the most common side effects [8]. Despite its potential, there is still uncertainty about the most appropriate intervention for alopecia areata, and more research is needed to establish minoxidil as a first-line treatment option [10].

We present the case of a 21-year-old female patient with drug-induced liver injury three weeks after initiating topical application of minoxidil.

## 2. Case Presentation

A 21-year-old female patient without significant medical history was referred for further evaluation of elevated liver enzymes. She initially presented to her primary care physician five days before admission with upper abdominal pain with preceding nausea and vomiting. Her stool was pale and pasty and her urine was very dark. Additionally, she experienced pollakisuria and pressure sensation during urination. The primary care physician prescribed pantoprazole (40 mg once daily), which she took for 4 days.

Upon hospital admission, she complained icteric sclera, followed by jaundice, pruritus, and dark urine. She denied fever, chills, diaphoresis, alcohol consumption, mushrooms, illicit drug use (including intravenous drugs), unusual food consumption, or recent sexual contacts outside her stable relationship. She also denied foreign travel or consumption of wild plants or mushrooms. Apart from contact with 2 dogs, she had no other animal exposures. The patient works as a florist but reported no contact with toxic or unknown plants in her workplace.

The patient reported having used a hair growth treatment prior to hospital admission due to hair loss. On examination, there was a single, oval-shaped area of hair loss measuring approximately 3 cm by 2 cm, with intact, nonirritated skin and no signs of lesions, inflammation, or exudation.

The patient had a history of Hashimoto's thyroiditis and had been taking L-thyroxin (50 μg) and for 3 years oral contraceptives (levonorgestrel 0.01 mg/ethinylestradiol 0.02). She suffered from alopecia areata, for which she had begun applying a minoxidil-containing preparation (minoxidil, 20mg/mL in an ethanol/propylene glycol solution, applied to the affected areas of the scalp 2 times daily, 1 mL per application, as recommended by the manufacturer) three and a half weeks prior to hospital admission. She was not diabetic (her BMI was 25.1 kg/m^2^), and she had no history liver disease. The patient did not report the use of any dietary supplements or anabolic androgenic steroids or illicit drugs.

Physical examination upon admission revealed stable cardiopulmonary condition and without neurological symptoms. There were no signs of hepatic encephalopathy. She had mucocutaneous jaundice and icteric sclera without any other cutaneous manifestations of liver disease. Abdominal examination revealed no pathological findings.

Liver ultrasound revealed a normal-sized liver without focal or diffuse lesions and no signs of pre-existing fatty liver disease or cirrhosis. Perfusion of all hepatic vessels was normal. Bile ducts were not dilated, and the gallbladder was normal without gallstones.

Liver enzyme activity was markedly elevated, as well as bilirubin: Total bilirubin is 284 μmol/L (normal range < 15 μmol); direct bilirubin is 232 μmol/L (normal range < 5 μmol); alanine aminotransferase (ALT) is 2742 U/L (normal range < 35 U/L); aspartate aminotransferase (AST) is 907 U/L (normal range < 35 U/L); alkaline phosphatase (AP) is 138 U/L (normal range 35–104 U/L); gamma-glutamyltransferase (gGT) is 45 U/L (normal range < 40 U/L); lactate dehydrogenase (LDH) is 280 U/L (norm 135–214 U/L); pseudo-cholinesterase (CHE) is 2.86 kU/L (norm 4.2–11.2 kU/L). Moreover, the patient presented with deranged coagulation parameters, an international normalized ratio (INR) of 1.43 (norm 0.8–1.25), and antithrombin III (AT III) of 35% (norm 83%–128%). The thyroid values were as follows: thyrotropin (TSH) 0.18 µIU/mL (norm 0.27–4.2 µIU/mL), free thyroxine (ft4) 13.6 pmol/L; free triiodothyronine (ft3) 6.0 pmol/L. Infection parameters were mildly elevated (C-reactive protein (CRP), 8.1 (norm < 5 mg/L), leukocytes 10.3 × 10^9^/L (norm 4–9 × 10^9^/L). There was no evidence of an infectious etiology: negative serology for hepatitis A, B, C, and E; negative Herpes Consensus PCR test (HSV1, HSV2, HSV6, VZV, EBV, CMV). Coxiella, brucella, and leptospira serology results were negative. Stool samples analyzed for gastroenteritis pathogens (PCR) were negative for Noro-, Rota-, Adeno-, Astro-, and Sapovirus. TPHA and HIV screening tests were likewise negative. Serological tests were also negative for Campylobacter, Clostridioides difficile, Salmonella, Shigella, Yersinia enterocolitica, and Aeromonas. Calprotectin was tested positive in a stool sample (146 mg/kg, norm < 80 mg/kg). Autoimmune panel including antismooth muscle antibodies (ASMAs), antinuclear antibodies (ANAs), antimitochondrial antibodies (AMAs), and LKM1 antibodies was all unremarkable. No evidence of copper storage disease was detected in either the liver biopsy or blood analysis.

**Figure 1:**
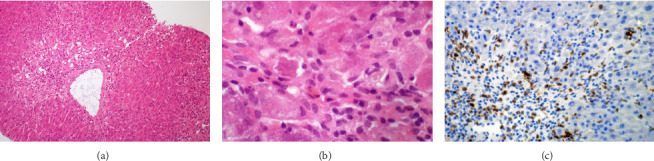
Liver biopsy showing (a) older centrilobular (zone 3) necrosis with disappearance of hepatocytes and granulation tissue around a central vein, original magnification × 100; (b) apoptotic body visible, 400x magnification. Both hematoxylin & eosin stain. (c) Immunohistochemistry for CD3 (T lymphocytes), 200x magnification.

Since laboratory tests, microbiological analyses, and ultrasound did not reveal a cause for the hepatic failure, a liver biopsy was performed. Histological examination revealed features of subacute liver dystrophy with older centrilobular necrosis (approximately 20% of the parenchyma), in zone 3 extending into zone 2, accompanied by nonspecific portal inflammation and mild cholestasis (Figure 1).

Pantoprazole, oral contraceptive, and minoxidil were discontinued upon admission. Due to impaired plasmatic coagulation, we administered 10 mg of phytomenadione intravenously, which led to normalization of the INR after 2 days. The mild hyperthyroidism without clinical manifestations prompted us to reduce the L-thyroxine dosage.

During hospitalization, transaminase levels declined without pharmacological treatment, and the clinical symptoms also improved. Upon discharge, only a minimal jaundice was still present, with no remaining skin discoloration. The patient was discharged after 9 days of hospitalization with markedly improved liver function (total bilirubin 120 μmol/L, INR 1.01, ALT 544 U/L, AST 160 U/L). Follow-up showed a further improvement of aminotransferases 7 days after discharge without any symptoms. 10 weeks after discharge, the liver values and thyroid hormones had normalized.

## 3. Discussion

This case presents an unusual case of severe drug-induced liver failure (Grade III, King's College Classification system [11]) in a young female patient following topical minoxidil application for alopecia areata, with comprehensive investigation systematically excluding alternative etiologies and identifying topical minoxidil as the most probable causative agent for the observed hepatic dysfunction.

The temporal relationship between minoxidil initiation and symptom onset (approximately three and a half weeks), along with the exclusion of other potential etiologies and improvement after medication discontinuation, strongly suggests a causal relationship according to the Roussel Uclaf Causality Assessment Method (RUCAM) criteria [12]. Only very few cases of minoxidil-induced liver have been reported. While DILI is commonly associated with herbal supplements, MDMA, cocain, antiepileptics, antituberculosis drugs, certain antidepressants, and topical minoxidil-induced hepatotoxicity are exceptionally rare [13]. While others have described elevated transaminases following topical minoxidil treatment, this represents—to the best of our knowledge—the first documented case of liver failure with impaired synthetic function attributed to topical minoxidil application.

To assess the likelihood of DILI in our patient, we applied the RUCAM scale, which yielded a score of 6, indicating a probable association between minoxidil use and liver injury. A higher causality score was not achieved due to the mixed hepatocellular and cholestatic pattern observed, and re-exposure to the suspected agent was unethical given the severity of the initial reaction [14].

Furthermore, the histopathologic findings of the liver show centrilobular necrosis that extended from zone 3 to zone 2, which is consistent with patterns observed in other cases of drug-induced hepatotoxicity [15, 16]. This pattern typically reflects exposure to hepatotoxic metabolites produced by cytochrome P450 enzymes in the centrilobular region [17]. While minoxidil is primarily metabolized hepatically by sulfotransferase enzymes and not by cytochrome P450 [18], the precise mechanisms by which its metabolites might cause hepatocellular damage require further investigation. Following exclusion of other causes, the present histological pattern is consistent with drug-induced liver injury.

Laboratory chemistry revealed markedly elevated transaminases (ALT 78x ULN (upper limit of normal), AST 26x ULN) and total bilirubin (284 μmol/L), combined with signs of impaired liver synthesis (INR 1.43, antithrombin III 35%). This severe liver enzyme increase distinguishes our case from previously reported cases of minoxidil induced hepatotoxicity, which typically appeared only as transaminase elevations without liver failure [19].

While topical application of minoxidil is often assumed to have minimal systemic absorption, the authors of [20] showed that topical minoxidil can lead to significant serum concentrations. This finding was confirmed by [21], who were able to detect minoxidil in the serum of seven out of 12 patients after topical application, with concentrations between 0.4 and 7.5 ng/mL. These studies show that even topically applied minoxidil has a systemic effect and can lead to potential hepatotoxicity.

Another study by [22] reported that a young woman developed elevated liver enzymes after an acute oral ingestion of topical minoxidil solution, mistaking it for cold medicine. Furthermore, a study evaluating side effects of low-dose oral minoxidil found mild transient elevations in liver function tests without significant clinical sequelae [15].

Although the patient was also treated with pantozole, an association with ALF is very unlikely even though PPI may cause toxic liver injury [19]. The patient had already developed symptoms characteristic for acute hepatic injury before starting pantoprazole, and the temporal relationship with minoxidil use (approximately three and a half weeks) is consistent with the typical latency periods observed in idiosyncratic DILI [17].

The pathophysiological mechanism of minoxidil-induced hepatotoxicity is not yet fully understood. Minoxidil was originally developed as an antihypertensive and acts mainly through vasodilation and activation of potassium channels [8]. Normally, systemic absorption of topical medications is minimal and ranges between 0.3% and 5.4% of the applied minoxidil dose [23, 24]. However, individual differences in dermal absorption and metabolism could play a significant role in rare idiosyncratic reactions [25]. For example, increased dermal absorption could be due to inflamed or damaged skin, which could be present in patients with alopecia areata due to underlying inflammatory processes [26].

The rapid clinical improvement of the presented case and laboratory regression of transaminases after discontinuation of the drug is characteristic of idiosyncratic DILI, which typically regresses as soon as the triggering drug is removed [27]. The absence of a rebound in transaminases or remission after discontinuation supports the diagnosis of DILI and not autoimmune hepatitis, which was further ruled out by negative autoimmune markers [25, 28].

Several factors may have contributed to the patient's increased susceptibility to this rare adverse event. The presence of Hashimoto's thyroiditis indicates an underlying autoimmunity, which has been associated with an increased risk of idiosyncratic drug reactions [16]. In addition, concomitant medications, including oral contraceptives with ethinylestradiol, may have altered liver metabolism or provided an additional trigger in the development of DILI [29]. Oral contraceptives, metabolized mainly via CYP3A4, may actually suppress rather than induce CYP3A4 activity [30–32]. However, the patient has been taking oral contraceptives for 3 years without hepatic complications, making the contraceptive less likely as the primary trigger. Minoxidil is primarily metabolized via sulfotransferases (SULT1A1/1A3) and glucuronidation to its active metabolite, minoxidil sulfate, with minimal involvement of CYP3A4 [33–35]. Given that minoxidil metabolism is largely independent of CYP3A4, a clinically relevant pharmacokinetic interaction is more unlikely. Hepatotoxicity occurred shortly after minoxidil initiation, resolved after its discontinuation, and persisted despite continued contraceptive use, supporting minoxidil as the likely cause.

Other toxic, infectious, or other potential causes of liver failure, including right heart failure, hepatic vein thrombosis, arterial circulatory disorders, autoimmune diseases such as autoimmune hepatitis, malignancies, and cholestatic conditions, were systematically excluded.

Patients with genetic polymorphisms in drug-metabolizing enzymes and human leukocyte antigen (HLA) alleles may be predisposed to idiosyncratic DILI [36]. This genetic testing was not performed in our patient, so we cannot rule out a genetic predisposition to develop DILI. Among other things, it could explain why only a small proportion of people exposed to the drug develop hepatotoxicity [37].

Our treatment approach was in line with current recommendations for DILI, which focus on immediate discontinuation of the suspected drug and supportive measures [25]. In addition, normalization of coagulation parameters after vitamin K dose and in the course of simply waiting and discontinuing the drug showed the regenerative capacity of the liver [38].

This case emphasizes the importance of considering all drug therapies, including topical application, when evaluating patients with unexplained hepatotoxicity. Medications that are generally considered minimally systemically absorbable should also be considered. Comprehensive medication histories, including over-the-counter drugs and topical preparations, remain essential in the diagnostic workup of acute liver injury [1].

In summary, this case report describes the first documented case of a severe hepatotoxic reaction with liver failure and impaired liver function attributable to topical minoxidil in a patient with alopecia areata. The case highlights the unpredictability of idiosyncratic drug reactions and emphasizes the need for attention when prescribing drugs with even minimal systemic absorption. Further research is needed to evaluate the mechanisms of minoxidil induced hepatotoxicity and to identify potential risk factors that may predispose certain individuals to this rare adverse event.

## Data Availability

All data generated or analyzed during this study are included in this article. Further enquiries can be directed to the corresponding author.
